# Identifying Content-Based Engagement Patterns in a Smoking Cessation Website and Associations With User Characteristics and Cessation Outcomes: A Sequence and Cluster Analysis

**DOI:** 10.1093/ntr/ntab008

**Published:** 2021-01-12

**Authors:** Olga Perski, Noreen L Watson, Kristin E Mull, Jonathan B Bricker

**Affiliations:** 1 Department of Behavioural Science and Health, University College London, London, UK; 2 Fred Hutchinson Cancer Research Center, Seattle, WA; 3 Department of Psychology, University of Washington, Seattle, WA

## Abstract

**Introduction:**

Using WebQuit as a case study, a smoking cessation website grounded in Acceptance and Commitment Therapy, we aimed to identify sequence clusters of content usage and examine their associations with baseline characteristics, change to a key mechanism of action, and smoking cessation.

**Methods:**

Participants were adult smokers allocated to the WebQuit arm in a randomized controlled trial (*n* = 1,313). WebQuit contains theory-informed content including goal setting, self-monitoring and feedback, and values- and acceptance-based exercises. Sequence analysis was used to temporally order 30-s website usage segments for each participant. Similarities between sequences were assessed with the optimal matching distance algorithm and used as input in an agglomerative hierarchical clustering analysis. Associations between sequence clusters and baseline characteristics, acceptance of cravings at 3 months and self-reported 30-day point prevalence abstinence at 12 months were examined with linear and logistic regression.

**Results:**

Three qualitatively different sequence clusters were identified. “Disengagers” (576/1,313) almost exclusively used the goal-setting feature. “Tryers” (375/1,313) used goal setting and two of the values- and acceptance-based components (“Be Aware,” “Be Willing”). “Committers” (362/1,313) primarily used two of the values- and acceptance-based components (“Be Willing,” “Be Inspired”), goal setting, and self-monitoring and feedback. Compared with Disengagers, Committers demonstrated greater increases in acceptance of cravings (*p* = .01) and 64% greater odds of quit success (OR_adj_ = 1.64, 95% CI = 1.18, 2.29, *p* = .003).

**Discussion:**

WebQuit users were categorized into Disengagers, Tryers, and Committers based on their qualitatively different content usage patterns. Committers saw increases in a key mechanism of action and greater odds of quit success.

**Implications:**

This case study demonstrates how employing sequence and cluster analysis of usage data can help researchers and practitioners gain a better understanding of how users engage with a given eHealth intervention over time and use findings to test theory and/or to improve future iterations to the intervention. Future WebQuit users may benefit from being directed to the values- and acceptance-based and the self-monitoring and feedback components via reminders over the course of the program.

## Introduction

Tobacco smoking is the leading cause of ill-health and premature death, with 8 million people globally dying of a smoking-related disease every year.^1^ Smokers’ chances of quitting are substantially increased with the use of pharmacological or behavioral support^2,3^; however, the majority of smoking cessation attempts are unaided.^4^ In the United States (US), this is partly explained by low accessibility to cessation support, particularly for disadvantaged smokers (e.g., smokers with lower socioeconomic status or who live in rural areas).^5^ With technological advancements, smokers have free access to effective cessation support via text messages, websites, and smartphone applications.^6^ In the US and the United Kingdom (UK), internet use is high among smokers at approximately 80%–86%.^7^ Although data from randomized controlled trials (RCTs) of web-based smoking cessation interventions suggest that they can help people quit,^6^ average follow-up and quit rates tend to be suboptimal at approximately 34% and 9%, respectively; there is hence room for improvement.

Engagement with web-based smoking cessation interventions, defined as the amount (e.g., time spent), depth (e.g., the number and type of features accessed) and frequency (e.g., the number of logins) of intervention usage,^8^ is a strong predictor of data retention in clinical trials (critical for internal validity) and successful behavior change.^8,9^ Engagement is considered an indicator of users’ exposure to critical, theory-informed intervention content.^10,11^ eHealth interventions generate vast amounts of automatically recorded usage data, yet researchers rarely take account of the comprehensive data captured.^11,12^ For example, log file records can provide insight into how individual users engaged with particular intervention content over time, including the order in which the content was accessed and at what frequency and duration.^11^ Such data can help address questions as to whether particular content-based patterns of engagement (“sequence clusters”) are related to key outcomes of interest. Importantly, the application of sequence and cluster analysis to usage data from eHealth interventions may help evaluate theory and inform future program design iterations.^13,14^

First, identifying content-based patterns of engagement that are linked to outcomes of interest can help empirically test the theory at hand.^13^ Intervention components are often developed with a view to engendering change to specific, theoretical mechanisms of action (i.e., psychological, physical or social processes that are hypothesized to catalyze behavior change).^15,16^ Examining whether patterns of engagement involving particular intervention components (e.g., the order, frequency, or duration of use) is associated with change to hypothesized theoretical mechanisms of action and smoking cessation can hence serve as a useful theory test. For example, change to a specific mechanism of action may occur without participants having accessed key content (or vice versa), and smoking cessation may occur without change to the postulated theoretical mechanism of action (or vice versa). If so, such evidence can help inform theory or intervention content revision.^13^

Second, detailed analysis of how subgroups of users engage with eHealth interventions over time can help inform future program design iterations and/or content tailoring decisions for new users. For example, if a subgroup of users who exhibit a particular pattern of engagement is found to have improved cessation outcomes, and that pattern of engagement is associated with certain psychological or sociodemographic characteristics, results may be useful for informing tailoring decisions for new users with similar psychological (e.g., commitment to quitting) or sociodemographic (e.g., age, educational level) characteristics. This is sometimes referred to as a “warm start” (i.e., using data from existing users to better tailor content for new users).^14,17^ Previous studies of web-delivered smoking cessation interventions have categorized users on the basis of their frequency of logins into, for example, “stable engagers” and “stable non-engagers”.^18–20^ However, no study to date has applied sequence and cluster analysis to determine similarities and differences in temporal, content-based engagement trajectories and associations with baseline characteristics, theoretical mechanisms of action, and smoking cessation.

WebQuit is a web-delivered smoking intervention based on Acceptance and Commitment Therapy (ACT), which posits that psychological flexibility is underpinned by six core processes: acceptance (i.e., actively embracing thoughts and feelings without attempting to change their frequency or form), cognitive defusion (i.e., reducing the tendency to treat thoughts as literal or absolute), being present (i.e., ongoing, non-judgmental contact with psychological events), self as context (i.e., fostering awareness of one’s flow of experiences without attachment to them), values (i.e., choosing purposive action and life directions consistent with important values) and committed action (i.e., progressively developing greater and greater patterns of action linked to one’s values).^21–23^ WebQuit includes theory-informed content, including values- and acceptance-based components (hypothesized to influence the acceptance of cravings to smoke), goal setting, self-monitoring, and feedback on progress (hypothesized to influence committed action to remaining smoke free), and an anonymous forum where users can submit questions to a stop smoking expert. A two-arm, stratified, double-blind RCT of WebQuit compared with the US Smokefree.gov website (*N* = 2,637) found similar 30-day point prevalence abstinence rates at 12 months across study arms (24% and 26%, respectively).^24^ A secondary data analysis found that WebQuit users categorized as “high engagers” (on the basis of a single engagement indicator—logins) saw larger increases in the acceptance of physical cravings at a 3-month follow-up (the only measured theoretical mechanism of action of the intervention) and increased chances of quit success at a 12-month follow-up compared with “low engagers.” ^24^ In a secondary analysis of the total duration of engagement with WebQuit, three distinct groups were identified, with 5- and 52-week users having greater odds of quit success compared with 1-week users.^25^ However, we currently lack knowledge as to how users engage with WebQuit’s features over time and in what order, and whether particular temporal, content-based engagement trajectories are associated with improved cessation outcomes. Although analyses of single engagement indicators (e.g., the frequency of logins) are perhaps considered more efficient than those combining multiple indicators, the former are not suited to addressing whether engagement with particular content over time is associated with outcomes of interest. The present study aimed to act as a case study, applying sequence and cluster analysis to usage data from WebQuit, to highlight how detailed analysis of usage data from eHealth interventions can help researchers, practitioners, and developers test theory and/or generate recommendations for future program design iterations. Specifically, this study had the following objectives:

1. To identify engagement sequence clusters (i.e., clusters of users with similar temporal and content-based patterns of engagement) in WebQuit.
2. To examine whether baseline user characteristics predict sequence cluster membership.
3. To examine whether sequence cluster membership predicts improvement in acceptance of cravings (a key theoretical mechanism of action) at a 3-month follow-up.
4. To examine whether sequence cluster membership predicts smoking cessation at a 12-month follow-up.

## Methods

### Study Design

We conducted a sequence and cluster analysis of usage data from participants allocated to the intervention arm (WebQuit) in an RCT of two web-delivered smoking cessation interventions.

### Study Setting and Population

As described elsewhere,^24^ participants (*N* = 2,637) from across the US were invited to participate in a study comparing two web-delivered smoking cessation interventions. Participants were recruited between March 2014 and August 2015. To be eligible to take part, participants had to be aged 18+ years, reside in the US, smoke at least 5 cigarettes per day (including dual cigarette smokers and e-cigarette users, but not those who were e-cigarette only users), be motivated to quit in the next 30 days and have internet access. The present study used data from participants assigned to the WebQuit arm (*n* = 1,319). To be included in the analytic sample, participants had to have accessed the website at least once in the first three months since study enrollment (*n/N* = 1,313/1,319).

### Intervention

**Figure 1.**
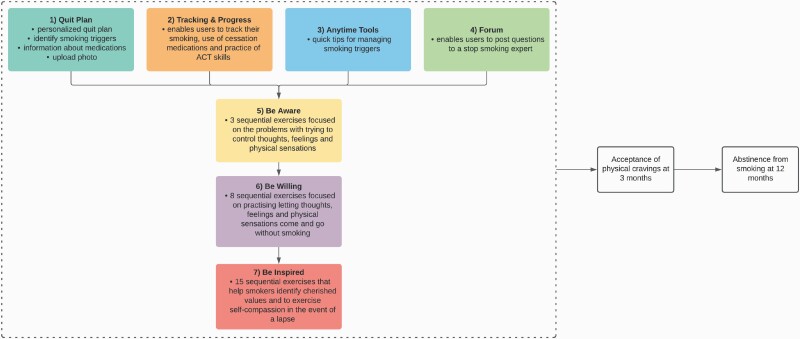
Map and conceptual model of WebQuit. It is hypothesized that engagement with WebQuit’s 7 components leads to increased acceptance of physical cravings at 3 months and improved odds of abstinence from smoking at 12 months. Components 1–4 are available at once; components 5–7 are sequentially unlocked. The color for each component maps onto the colors in Figures 2 and 3.

Participants were free to use WebQuit for 1 year from the date of enrollment. WebQuit is grounded in ACT, and teaches smokers skills to be willing to experience cravings as and when they occur without smoking.^23^ WebQuit consists of seven distinct components (see Figure 1), four of which are immediately available as and when needed by the user and three are sequentially unlocked (i.e., the subsequent components are only available after completion of the immediately preceding component) and then available as and when needed: (1) “Quit Plan,” which enables users to set up a personalized quit plan (i.e., goal setting), identify smoking triggers, learn about medications approved by the US Food and Drug Administration and upload a photo of their inspiration to quit; (2) “Tracking & Progress,” which enables users to track their smoking, use of cessation medications and practice of ACT skills over the course of the program (i.e., self-monitoring and feedback); (3) “Anytime Tools,” which provides quick tips for managing four different types of smoking triggers (i.e., those triggered by places, people, activities, and one’s own body/mind); and (4) “Forum,” which enables users to pose smoking-related questions to a stop smoking expert; (5) “Be Aware,” which contains three sequentially unlocked exercises that help illustrate the problems with trying to control thoughts, feelings, and physical sensations (as opposed to allowing them to come and go); (6) “Be Willing,” which contains eight sequentially unlocked exercises that help users practice allowing thoughts, feelings, and physical sensations that trigger smoking to come and go without smoking; and (7) “Be Inspired,” which contains 15 sequentially unlocked exercises that help participants identify cherished values inspiring them to quit smoking and to exercise self-compassion in the event of a smoking lapse.

### Measures

#### Baseline Characteristics

Participants provided baseline data on demographic (e.g., age, gender, ethnicity), smoking (e.g., nicotine dependence, number of friends who smoke), physical (e.g., self-rated health status) and mental health (e.g., depression, anxiety) characteristics (see Table 1).

**Table 1.** Variables Assessed at Baseline

**Demographic characteristics** • Age (continuous) • Gender (female, male) • Ethnicity (White, Black/African American, Hispanic) • Married^26^ (no, yes) • Lesbian/gay/bisexual^27^ (no, yes) • Working (no, yes) • High school education or less (no, yes) • Low family income, defined as <$20,000/year (no, yes) **Smoking characteristics** • Nicotine dependence, measured with the 6-item Fagerström Test for Nicotine Dependence (FTND) (range: 0–10)^28^ • Smoking half a pack a day or more (no, yes) • >10 years of smoking (no, yes) • Number of quit attempts in the past 12 months (continuous) • Number of friends who smoke (continuous) • Partner who smokes (no, yes) • Any use of e-cigarettes in the past 30 days (no, yes) • Commitment to quitting smoking, measured with a modified, 11-item version of the Commitment to Quitting Scale (range: 1–5)^29^ • Acceptance of physical cravings, measured with the 9-item “Physical Sensations” subscale of the Avoidance and Inflexibility Scale (range: 1–5), adapted from Refs. [^30,31]^ **Mental health characteristics** • Hazardous alcohol consumption, defined as ≥4 drinks per day for women and ≥5 drinks per day for men (no, yes) • Depression, measured with the Center for Epidemiologic Studies-Depression (CES-D) Scale, with scores of ≥16 classified as screening positive^32^ (no, yes) • Anxiety, measured with the General Anxiety Disorder-7 (GAD-7) Scale, with scores of ≥10 classified as a positive screen^33^ (no, yes) • Panic, measured with the Autonomic Nervous System Questionnaire, with those reporting ≥one panic attack within the past month that occurred in a situation in which they were not in danger or the center of attention classified as a positive screen^34^ (no, yes) • Post-traumatic stress disorder (PTSD), measured with the PTSD Checklist, with scores of ≥14 classified as screening positive^35^ (no, yes) • Social anxiety, measured with the Mini-Social Phobia Inventory, with scores of ≥6 indicating a positive screen^36^ (no, yes) • Self-reported presence of bipolar disorder (no, yes), schizophrenia (no, yes), and drug abuse (no, yes), measured with a single item developed by the North American Quitline Consortium (“Do you have any of the following mental health conditions?”)^37^ **Physical health characteristics** • Mobility (i.e., problems in walking about), self-care (i.e., problems washing oneself), the ability to perform usual activities (e.g., work, study, housework) and experience of bodily pain/discomfort, measured with the EuroQol EQ-5D Questionnaire (range: 1–5)^38^ • Self-rated health status (range: 0–100), measured with a single item (“How good is your health today?”)

#### Engagement Sequence Clusters

For each participant, a time- and date-stamped log file record was made each time they opened a new WebQuit page from enrolment to the 3-month follow-up assessment. Log files were sorted into the components described above: “Quit Plan” (i.e., goal setting), “Tracking & Progress” (i.e., self-monitoring and feedback), “Anytime Tools,” “Forum,” “Be Aware,” “Be Willing,” and “Be Inspired” (i.e., values- and acceptance-based exercises). Log file records of the opening of pages without any smoking-related content (e.g., help pages, settings) were categorised as “Other.” For each participant, the tagged log files were transformed into an ordered sequence of 30-s “active” engagement segments (i.e., de-fragmenting time spent on the program, removing inactive segments) for further analysis.^39^ Hence, calendar time (or total duration of WebQuit use in days) was no longer a dimension of the dataset. Log file records of pages where participants spent <30 s were excluded and those where participants spent >300 s (or 5 min) were truncated to 30-s segments, as such lengths were judged to be indicative of the participant having logged off the website (which is not explicitly recorded within WebQuit). The empirical cumulative distribution function indicated that 86% of all page views fell below the selected 300-s upper limit (see Supplementary file 1). Sensitivity analyses were performed with alternative cut-offs for the lower page view limit set to 15 and 5 s, respectively (see Supplementary file 1). The point at which 85% of participants had stopped engaging with the program was used as the final cut-off, selected for pragmatic reasons (i.e., to prevent the cluster analysis from focusing too heavily on engagement vs. disengagement, as opposed to the pattern and order of content used). This occurred when participants had spent a total of 50 active minutes (or 3,000 s) on WebQuit, or at the 100th 30-s segment. Sensitivity analyses were performed with alternative cut-offs set to 75%, 50%, and 25%, respectively (see Supplementary file 1).

#### Acceptance of Physical Cravings (Theoretical Mechanism of Action)

Change in acceptance of physical cravings at the 3-month follow-up was measured by subtracting the 3-month follow-up score from the baseline score on the “Physical Sensations” subscale of the Avoidance and Inflexibility Scale.^31^ To reduce participant burden, this was the only theoretical mechanism of action assessed in the RCT.

#### Smoking Cessation

Self-reported 30-day point prevalence abstinence (i.e., no smoking at all in the past 30 days) was measured at the 12-month follow-up. Two analyses were planned, the first included participants who completed the 12-month follow-up survey (i.e., complete-case analysis) and the second included all participants, with those missing 12-month data assumed to be smokers (i.e., imputed missing-equals-smoking analysis). However, due to observed differential attrition across clusters that could systematically bias analyses in favor of groups with less missingness^40^ and as a result of the review process, we decided to remove the missing-equals-smoking analysis.

### Data Analysis

The analyses were conducted in R v.3.6.3 using the *TraMineR* package, specifically developed to facilitate the description and analysis of discrete sequence data.^41^

First, sequence analysis was used to visualize the temporally ordered sequences of 30-s engagement segments for each individual. Similarities between sequences were statistically assessed with the optimal matching (OM) distance algorithm.^41^ The OM algorithm calculates a distance measure for each pair of sequences in the dataset, which represents the degree of temporal and content-based similarity between any given pair. The pairwise distances were subsequently used as input for an agglomerative hierarchical clustering analysis, which was used to determine whether meaningful groupings could be constructed.^42^ The “elbow method” was used to determine the number of sequence clusters to retain.^43^

Second, baseline characteristics that predict sequence cluster membership were assessed using a multivariable multinomial logistic regression analysis. Variables that were independently associated (*p* < .05) with the sequence clusters in univariable logistic regression analyses were included in the subsequent multivariable analysis. Third, a linear regression analysis was used to assess whether sequence cluster membership predicts change in the acceptance of cravings at the 3-month follow-up, adjusting for baseline characteristics that significantly predict cluster membership. Fourth, a logistic regression analysis was performed to examine whether sequence cluster membership predicts quit success at the 12-month follow-up, adjusting for baseline characteristics that significantly predict cluster membership. Participants with missing data on the variables of interest were excluded from the inferential analyses.

## Results

### Participant Characteristics

Table 2 shows participants’ baseline demographic, mental health, smoking, and physical health characteristics. Participants were on average 46.2 years old, 79.4% female, 73.5% White, and 27.5% reported a low family income (<$20,000/year).

**Table 2.** Participants’ Baseline Characteristics (*N* = 1,313)

Baseline characteristics	
Demographic characteristics	
Female, % (*n*)	79.4% (1,043)
Age, mean (SD)	46.2 (13.4)
White ethnicity, % (*n*)	73.5% (965)
Black/African American ethnicity, % (*n*)	10.3% (135)
Hispanic ethnicity, % (*n*)	7.7% (101)
Married, % (*n*)	39.5% (518)
Lesbian/Gay/Bisexual, % (*n*)	8.8% (116)
Working, % (*n*)	53.0% (696)
High school or less, % (*n*)	28.0% (367)
Low family income, % (*n*)^a^	27.5% (361)
Mental health characteristics	
Screened positive for depression, % (*n*)^b^	55.6% (730)
Screened positive for anxiety, % (*n*)^c^	33.1% (435)
Screened positive for panic, % (*n*)^d^	42.6% (559)
Screened positive for PTSD, % (*n*)^e^	52.2% (686)
Screened positive for social anxiety, % (*n*)^f^	29.6% (388)
Screened positive for hazardous alcohol consumption, % (*n*)^g^	10.7% (140)
Self-reported bipolar disorder, % (*n*)	8.1% (107)
Self-reported schizophrenia, % (*n*)	0.8% (10)
Self-reported drug use, % (*n*)	2.6% (34)
Smoking characteristics	
FTND, mean (SD)	5.6 (2.2)
Half a pack or more, % (*n*)	79.3% (1,041)
>10 years of smoking, % (*n*)	80.0% (1,050)
Quit attempts in past 12 months, mean (SD)^h^	1.7 (5.3)
Number of friends who smoke, mean (SD)	2.2 (1.6)
Partner who smokes, % (*n*)	30.3% (398)
Any use of e-cigarettes in the past 30 days, % (*n*)	34.4% (452)
Commitment to quitting, mean (SD)^i^	4.0 (0.7)
Acceptance of cravings, mean (SD)^j^	2.9 (0.5)
Physical health characteristics	
Mobility, mean (SD)	0.5 (0.8)
Self-care, mean (SD)	0.1 (0.4)
Usual activities, mean (SD)	0.5 (0.8)
Pain/discomfort, mean (SD)	1.1 (1.0)
Self-rated health status, mean (SD)^k^	70.8 (18.9)
Body mass index, mean (SD)^l^	29.2 (7.5)

^a^ Data missing for 1 participant.

^b^ Data missing for 6 participants.

^c^ Data missing for 12 participants.

^d^ Data missing for 140 participants.

^e^ Data missing for 7 participants.

^f^ Data missing for 4 participants.

^g^ Data missing for 31 participants.

^h^ Data missing for 77 participants.

^i^ Data missing for 7 participants.

^j^ Data missing for 17 participants.

^k^ Data missing for 2 participants.

^l^ Data missing for 8 participants.

#### Engagement Sequence Clusters

**Figure 2.**
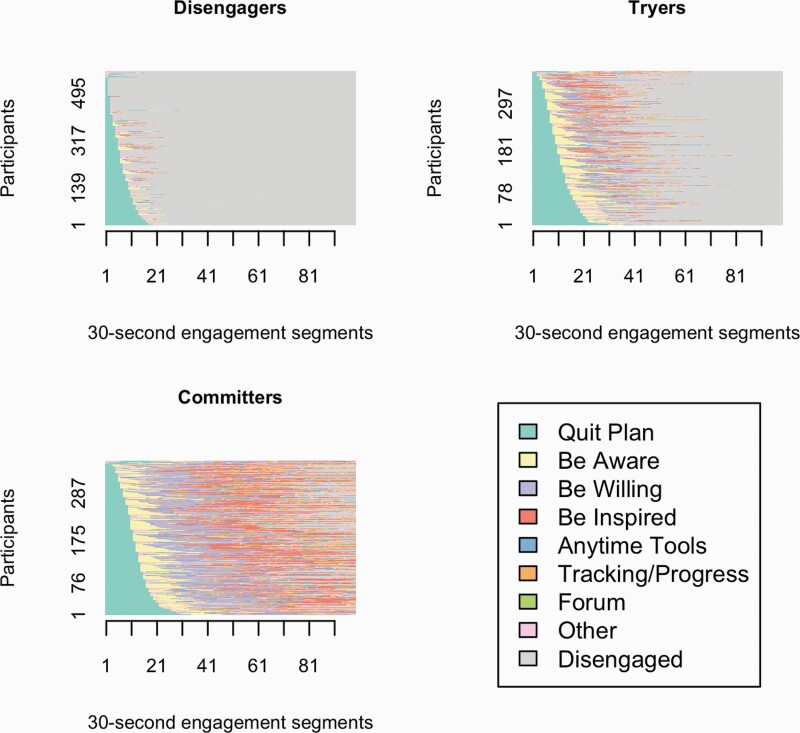
Plots of ordered 30-s engagement segments (*x*-axis) for participants (*y*-axis), stratified by sequence cluster. “Disengaged” (gray segments) indicate time when participants were no longer actively engaged with WebQuit (i.e., they had stopped logging into the website).

**Figure 3.**
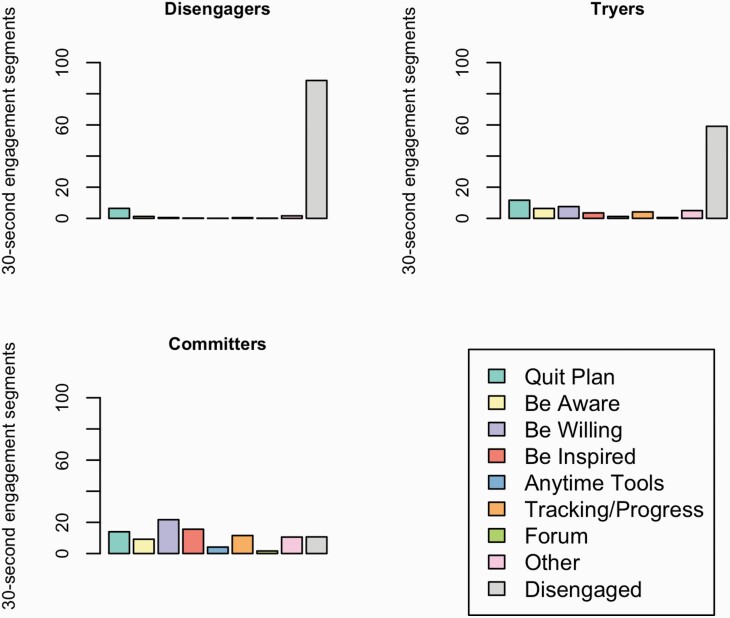
Plots of the average time spent within each WebQuit component, stratified by sequence cluster. “Disengaged” (gray bars) indicate time when participants were no longer actively engaged with WebQuit (i.e., they had stopped logging into the website).

Three qualitatively different engagement sequence clusters were identified. Figure 2 shows the ordered sequences of 30-s engagement segments for participants, stratified by engagement sequence cluster. Figure 3 shows the average time spent within each of WebQuit’s components, stratified by engagement sequence cluster. The “Disengagers” (576/1,313; 43.9%) almost exclusively engaged with the goal setting component (“Quit Plan”). The “Tryers” (375/1,313; 28.6%) primarily engaged with the goal setting (“Quit Plan”) and two of the values- and acceptance-based components (“Be Aware” and “Be Willing”). The “Committers” (362/1,313; 27.6%) primarily engaged with two of the values- and acceptance-based components (“Be Willing,” “Be Inspired”) and the goal setting (“Quit Plan”). However, they also engaged frequently with the self-monitoring and feedback component (“Tracking & Progress”) and a third values- and acceptance-based component (“Be Aware”). In a series of sensitivity analyses where alternative cut-offs were used, the cluster results were materially unchanged (see Supplementary file 1).

#### Baseline Predictors of Sequence Cluster Membership

Participants who had used e-cigarettes in the 30 days prior to the baseline assessment (OR = 1.70, 95% CI = 1.26, 2.29, *p* < .001) and had lower self-rated health status (OR = 0.99, 95% CI = 0.98, 0.99, *p* = .007) were more likely to be Tryers than Disengagers. Older participants (for each additional year of age, OR = 1.04, 95% CI = 1.02, 1.05, *p* < .001) and those who had used e-cigarettes in the past 30 days (OR = 1.49, 95% CI = 1.01, 1.89, *p* = .045) were more likely to be Committers than Disengagers. No other baseline characteristics were significant predictors of engagement sequence cluster membership (see Table 3).

**Table 3.** Multivariable Multinomial Logistic Regression Analysis Predicting Sequence Cluster Membership from the Baseline Characteristics

Baseline characteristics	Disengagers, % (*n*/*N*)	Tryers, % (*n*/*N*)	Committers, % (*n*/*N*)	Tryers vs. Disengagers, OR (95% CI)	*p*	Committers vs. Disengagers, OR (95% CI)	*p*
Female	76.2% (439/576)	80.5% (302/375)	83.4% (302/362)	1.23 (0.86, 1.74)	.25	1.44 (0.98, 2.12)	.06
Age, mean (SD)	43.3 (13.5)	46.5 (12.7)	50.5 (12.7)	1.01 (0.99, 1.03)	.10	1.04 (1.02, 1.05)	<.001
Employed	59.0% (340/576)	49.3% (185/375)	47.2% (171/362)	0.83 (0.61, 1.13)	.24	0.93 (0.68, 1.29)	.67
Screened positive for depression	58.0% (334/576)	56.8% (213/375)	50.6% (183/362)	1.20 (0.82, 1.75)	.35	0.91 (0.61, 1.36)	.64
Screened positive for anxiety	36.3% (209/576)	33.1% (124/375)	28.2% (102/362)	0.85 (0.59, 1.23)	.38	0.74 (0.50, 1.10)	.13
Screened positive for panic disorder	45.5% (262/576)	44.0% (165/375)	36.5% (132/362)	1.01 (0.74, 1.37)	.97	0.89 (0.64, 1.24)	.49
Screened positive for PTSD	54.7% (315/576)	52.0% (195/375)	48.6% (176/362)	0.88 (0.61, 1.28)	.51	1.13 (0.76, 1.67)	.55
Self-reported bipolar disorder	10.2% (59/576)	6.4% (24/375)	6.6% (24/362)	0.65 (0.38, 1.11)	.11	0.85 (0.48, 1.50)	.58
>10 years of smoking	76.0% (438/576)	79.7% (299/375)	86.5% (313/362)	0.83 (0.55, 1.25)	.38	0.93 (0.59, 1.46)	.74
Any e-cigarette use in the past 30 days	29.7% (171/576)	40.8% (153/375)	35.4% (128/362)	1.70 (1.26, 2.29)	< .001	1.38 (1.01, 1.89)	.045
Hazardous alcohol consumption	12.8% (74/576)	10.7% (40/375)	7.2% (26/362)	0.84 (0.54, 1.31)	.44	0.74 (0.45, 1.23)	.24
Mobility, mean (SD)	0.4 (0.8)	0.5 (0.9)	0.6 (0.9)	1.05 (0.82, 1.34)	.70	0.93 (0.72, 1.20)	.58
Self-care, mean (SD)	0.1 (0.3)	0.1 (0.4)	0.1 (0.5)	1.17 (0.74, 1.86)	.51	1.47 (0.94, 2.28)	.09
Usual activities, mean (SD)	0.5 (0.8)	0.5 (0.8)	0.7 (0.9)	0.87 (0.67, 1.12)	.28	1.19 (0.92, 1.54)	.19
Pain/discomfort, mean (SD)	1.0 (1.0)	1.1 (1.0)	1.2 (1.0)	0.99 (0.83, 1.20)	.99	0.99 (0.81, 1.20)	.90
Self-rated health status, mean (SD)	72.3 (17.8)	68.4 (20.3)	70.7 (18.9)	0.99 (0.98, 0.99)	.007	0.99 (0.99, 1.01)	.99

Variables included in the multivariable analysis were those with significant independent associations in univariable analyses. Data on the baseline characteristics of interest were missing for 181 participants.

#### Associations of Sequence Cluster Membership With Change to a Key Mechanism of Action and Smoking Cessation

The 12-month data retention rate was 87% (Disengagers: 82% (474/576); Tryers: 87% (328/375); Committers: 93% (335/362)). Compared with Disengagers, being in the Committers cluster was associated with a 0.14-point increase (on a 5-point scale) in the acceptance of cravings (*p* = .01). In a complete-case analysis, Committers had significantly greater odds of quit success compared with Disengagers (OR_adj_ = 1.64, 95% CI = 1.18, 2.29, *p* = .003; see Table 4).

**Table 4.** Linear and Logistic Regression Analyses of the Relationship Between Sequence Cluster Membership and Change in Acceptance of Cravings at 3 Months and Self-Reported Quit Success at 12 Months

Change in acceptance of cravings at 3 months					
	Mean change (SD)	B (95% CI)	*p*	Adjusted B (95% CI)*	*p*
Disengagers^a^	0.08 (0.61)	Ref	—	Ref	—
Tryers^b^	0.05 (0.67)	–0.03 (–0.13, 0.06)	.47	–0.02 (–0.12, 0.07)	.62
Committers^c^	0.20 (0.71)	0.12 (0.02, 0.21)	.02	0.14 (0.04, 0.23)	.01
Quit success at 12 months—complete case analysis					
	% (*n*)	OR (95% CI)	*p*	Adjusted OR (95% CI)*	*p*
Disengagers^d^	21.9% (104/474)	Ref	—	Ref	—
Tryers^e^	22.0% (72/328)	1.00 (0.71, 1.40)	.99	1.07 (0.75, 1.50)	.71
Committers^f^	30.1% (101/335)	1.54 (1.12, 2.11)	.01	1.64 (1.18, 2.29)	.003

^a^ Data were missing for 143 participants.

^b^ Data were missing for 53 participants.

^c^ Data were missing for 37 participants.

^d^ Data were missing for 102 participants.

^e^ Data were missing for 47 participants.

^f^ Data were missing for 27 participants.

* Adjusted for age, self-rated health status and any use of e-cigarettes in the past 30 days.

## Discussion

### Principal Findings

This case study in the application of sequence and cluster analysis to WebQuit users’ log file records identified three distinct and qualitatively different sequence clusters, which we labelled Disengagers, Tryers, and Committers. Participants who had used e-cigarettes in the past 30 days and rated their health status as lower were more likely to be categorised as Tryers than Disengagers, but Tryers did not show a differential change in the acceptance of cravings or likelihood of quitting smoking. Older participants and those who had used e-cigarettes in the past 30 days were more likely to be Committers than Disengagers, increased their acceptance of cravings at 3 months at a greater rate and had greater odds of quit success at 12 months.

### Identification of Content-Based Engagement Sequence Clusters

To our knowledge, this is the first study to identify sequence clusters on the basis of how individual users engaged with the content of an eHealth intervention for smoking cessation (but see refs. ^39,44–46^ for examples of the application of sequence and/or cluster analysis to usage data from eHealth interventions for weight loss, physical activity, and mental health). The key factor distinguishing Committers from Tryers is that the former primarily engaged with two of the values- and acceptance-based components (“Be Willing” and “Be Inspired”), in addition to the self-monitoring and feedback component; this is a plausible explanation as to why acceptance of cravings increased to a greater extent in Committers, who were also found to be more likely to quit smoking in the complete case analysis. It should, however, be noted that experimental (as opposed to observational) work where users are randomized to receive different combinations of components^47^ or delivery schedules is required for robust causal inference (i.e., pinpointing features/delivery schedules that are causally linked to smoking cessation outcomes).

### Baseline Predictors of Engagement Sequence Cluster Membership

The finding that older participants were more likely to be Committers than Disengagers echoes findings from the extant literature: older age has previously been found to predict greater levels of engagement with eHealth interventions for smoking cessation and alcohol reduction.^8,48^ It is unclear what aspect of someone’s age is relevant for how they engage with eHealth interventions, but younger adults may be less willing to return to content they have already been exposed to and open only to new content.^48^ The finding that e-cigarette use in the past 30 days at baseline predicted Tryer and Committer sequence cluster membership may be interpreted to suggest that the seeking out of quitting aids (in addition to the web-based program) is reflective of greater quitting self-efficacy, motivation to stop, or other unmeasured variables that are motivational in kind. This should be further explored in future research. The observation that few baseline characteristics predicted sequence cluster membership in the present study is unexpected and limits recommendations for how to better tailor WebQuit content for particular subgroups of users. A potential explanation for this observation is that factors other than baseline characteristics are important for predicting patterns of engagement, such as perceived usefulness of the program or successful craving management following early program exposure.^8,49^ The role of time-varying covariates in the prediction of user engagement hence merits further exploration.^49^

### Associations of Sequence Cluster Membership With Smoking Cessation Outcomes

Committers engaged with two of the values- and acceptance-based and the self-monitoring and feedback components more frequently and spent more time on these features compared with Disengagers and Tryers, and also had greater improvements in acceptance of cravings at 3 months and greater odds of quit success at 12 months. First, this finding can be interpreted to suggest that principles from Acceptance and Commitment Therapy are effective in helping smokers increase their acceptance of cravings to smoke. Second, it may also be interpreted to suggest that future WebQuit users would benefit from being directed to the values- and acceptance-based and the self-monitoring and feedback components over the course of the program, for example through e-mail reminders or notifications to return to such exercises on a regular basis. The use of sequence and cluster analysis to tailor feature delivery schedules or messages to prompt the use of particular features merits further investigation. It may, however, be useful to focus on proximal outcomes (e.g., acceptance of cravings after 1 week rather than 3 months) to allow for quicker iteration of novel design features, in line with intervention optimization methods guided by the Multiphase Optimization Strategy.^50,51^

#### Limitations

This study had several limitations. First, there were no clear mappings of WebQuit components to broader content categories (e.g., the Quit Plan contained both goal setting and information about approved stop smoking medications) and no new content was available to users after they completed the core WebQuit program. Hence, findings may not generalize to other interventions that are structured differently. Second, the analytic approach opted for here meant that calendar time was not a dimension of our dataset; this further limits the generalizability of the results to different settings, interventions, or populations. For example, Committers could have engaged frequently with the values- and acceptance-based components within the first month of the program (rather than consistently throughout the 3-month period used as cut-off for the current study). Therefore, specific recommendations as to the time period required for particular content usage to influence key mechanisms of action cannot be provided based on the current analysis. Although it would have been technically possible to chunk engagement segments according to calendar time (e.g., hours, days, weeks in the program)—which is standard practice in the life course literature where sequence and cluster analysis is frequently applied^52,53^—we deemed it more appropriate to standardize participants’ diverse time series into “active” engagement segments. Future research should explore different ways of chunking usage data from eHealth interventions and examine the impact on sequence and clustering results. Third, although sequence and cluster analysis can account for the order in which users access different program features, we were unable to distinguish order from frequency/amount of use in the present case study, with the identified clusters also mapping onto low, medium and high engagement groups at the surface level. However, when looking more closely at the specific content used, a qualitative difference in usage patterns by cluster membership was observed. It is, however, plausible that the sequential structure of the WebQuit program may have limited variability in content usage. In an eHealth intervention for physical activity where content was delivered all at once, researchers were able to identify 3 types of usage sessions (as opposed to user types) on the basis of the probabilities of moving from one page to another.^45^ Probabilistic approaches, such as Markov Chain analysis, may hence be more fruitful for identifying content-based patterns of engagement in eHealth interventions. Future research should conduct a head-to-head comparison of available approaches and across different use cases before drawing any firm conclusions as to the utility of sequence and cluster analysis. Fourth, although response to the 12-month follow-up survey was high at 87%, there was differential attrition (range: 82%–93%) across the identified engagement clusters. As the Type I error risk is high in missing-equals-smoking analyses where there is differential attrition across conditions, favoring the arm(s) with a higher response rate,^40^ we decided only to present results from a complete-case analysis. This speaks to the well-established finding that low intervention engagement is also tied to the provision of follow-up data in eHealth interventions.^54^

## Conclusions

This case study demonstrates how employing sequence and cluster analysis can help researchers and practitioners gain a better understanding of how users engage with a particular eHealth intervention over time and use findings to test theory and/or to improve feature delivery schedules for new subgroups of users. In this context, WebQuit users were categorised into Disengagers, Tryers, and Committers based on their patterns of content usage. Committers saw increases in a key theoretical mechanism of action at 3 months and increased odds of quit success at 12 months. Future WebQuit users may benefit from being directed to the values- and acceptance-based exercises and self-monitoring and feedback features via reminders over the course of the program.

## Supplementary Material

A Contributorship Form detailing each author’s specific involvement with this content, as well as any supplementary data, are available online at https://academic.oup.com/ntr.

ntab008_suppl_Supplementary_File

ntab008_suppl_Supplementary_Taxonomy_Form
