# Supplementary material for: Identifying Content-Based Engagement Patterns in a Smoking Cessation Website and Associations With User Characteristics and Cessation Outcomes: A Sequence and Cluster Analysis
Source: Nicotine Tob Res. 2021 Jan 12;23(7):1103–12. doi: 10.1093/ntr/ntab008 (PMC8186423; doi:10.1093/ntr/ntab008)
Supplement: ntab008_suppl_Supplementary_File [file ntab008_suppl_supplementary_file.docx]

**Supplementary file 1**

***Sensitivity analyses for the engagement sequence clusters with alternative cut-offs***

*a) Empirical cumulative distribution function (ECDF) displaying the cumulative distribution of time spent per page (in seconds) in the total sample*


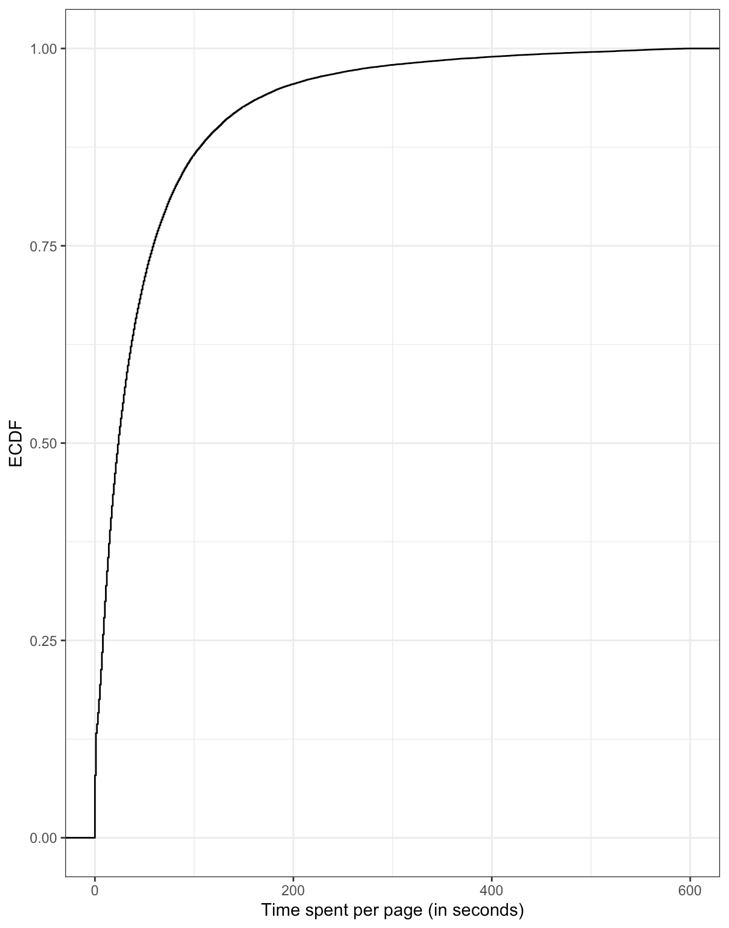


*b) Log file records of pages where participants spent <15 seconds excluded*


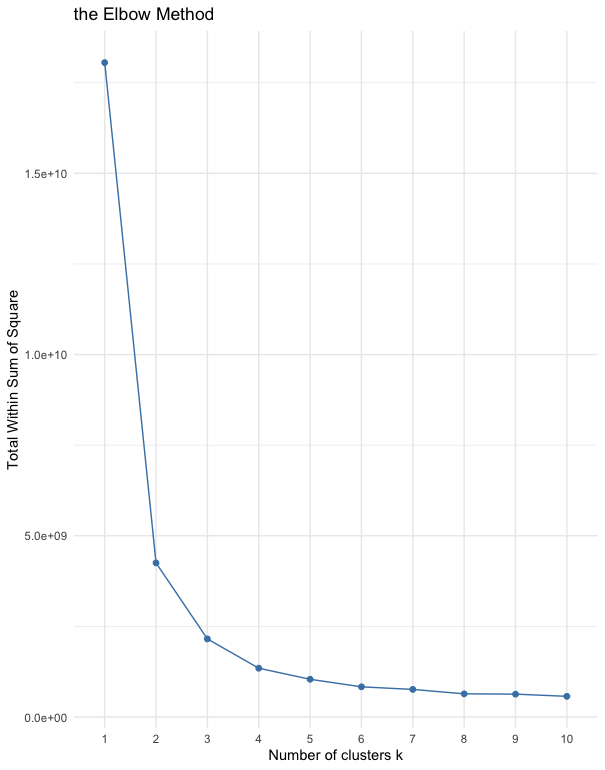


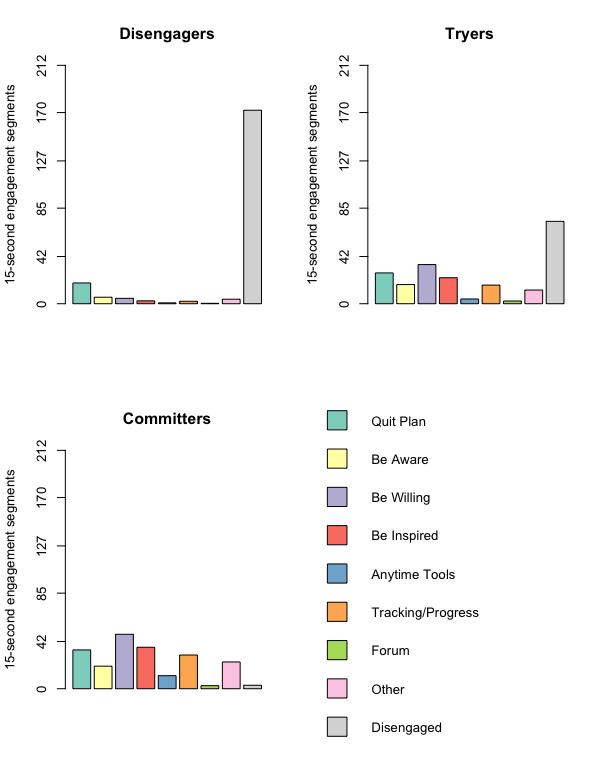


*c) Log file records of pages where participants spent <5 seconds excluded*

*
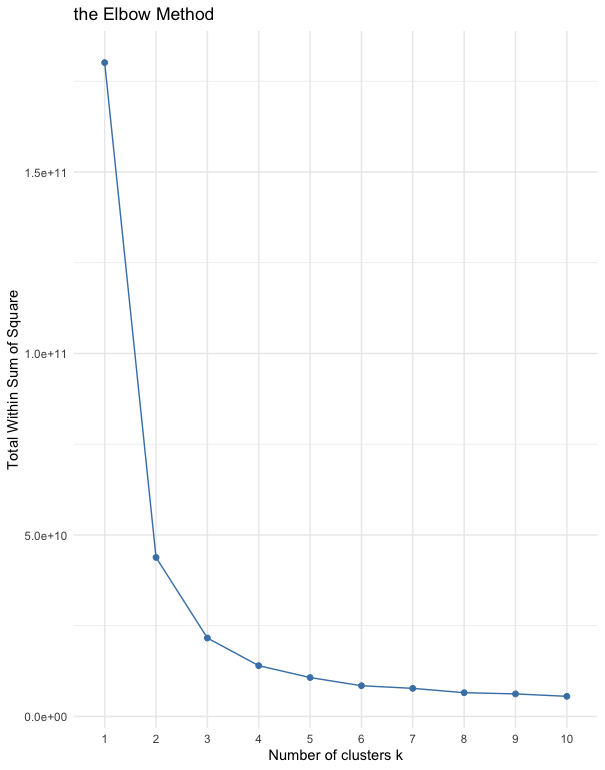
*

*
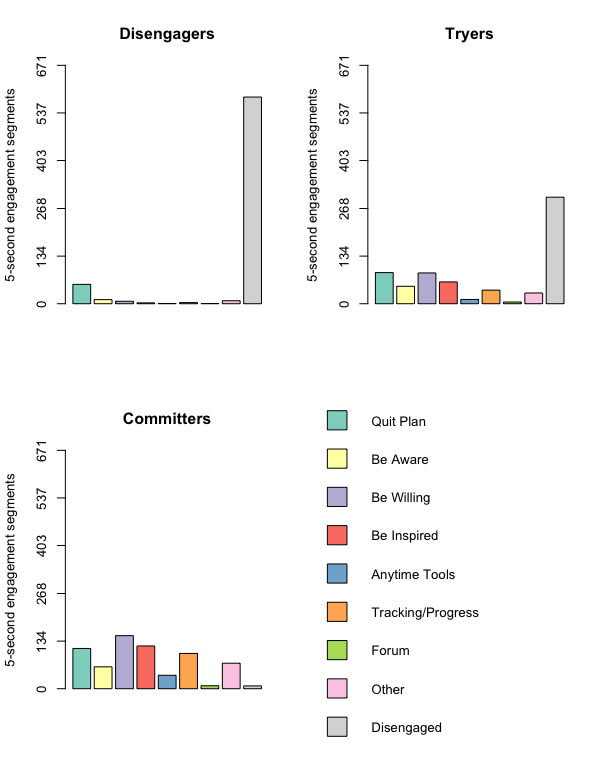
*

*d) The point at which 75% of participants had stopped engaging with the program used as cut-off*

*
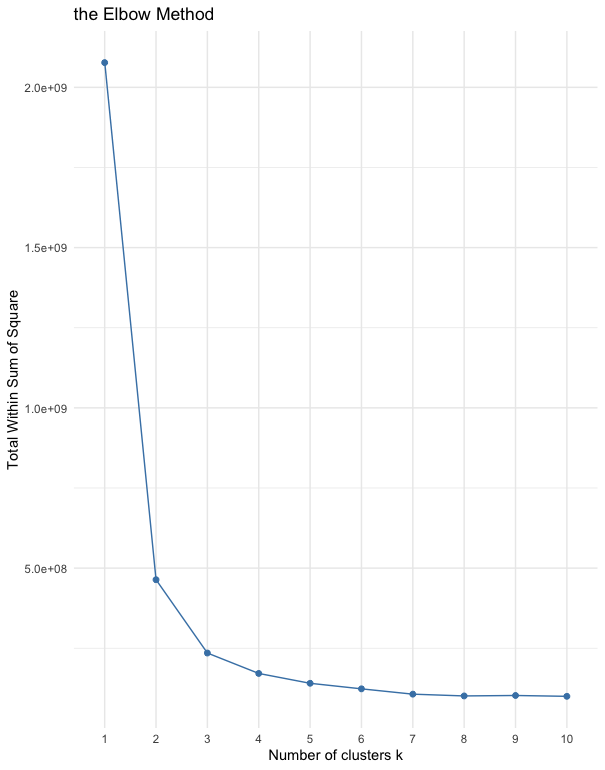
*

*
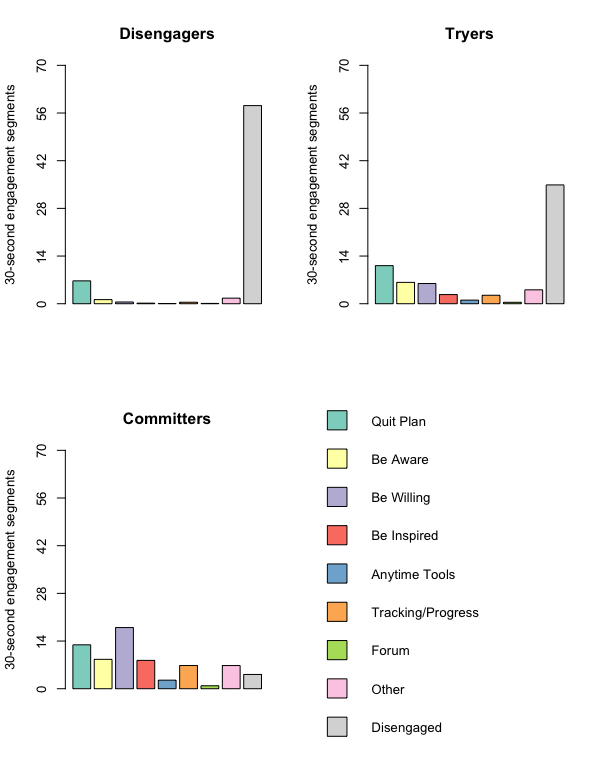
*

*e) The point at which 50% of participants had stopped engaging with the program used as cut-off*

*
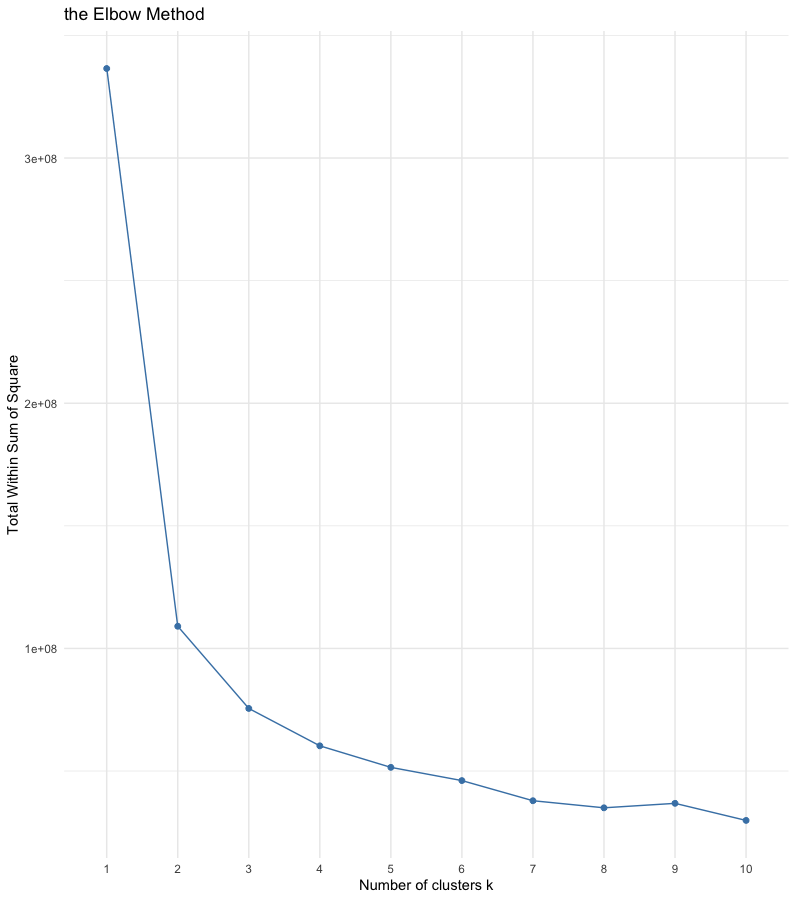
*

*
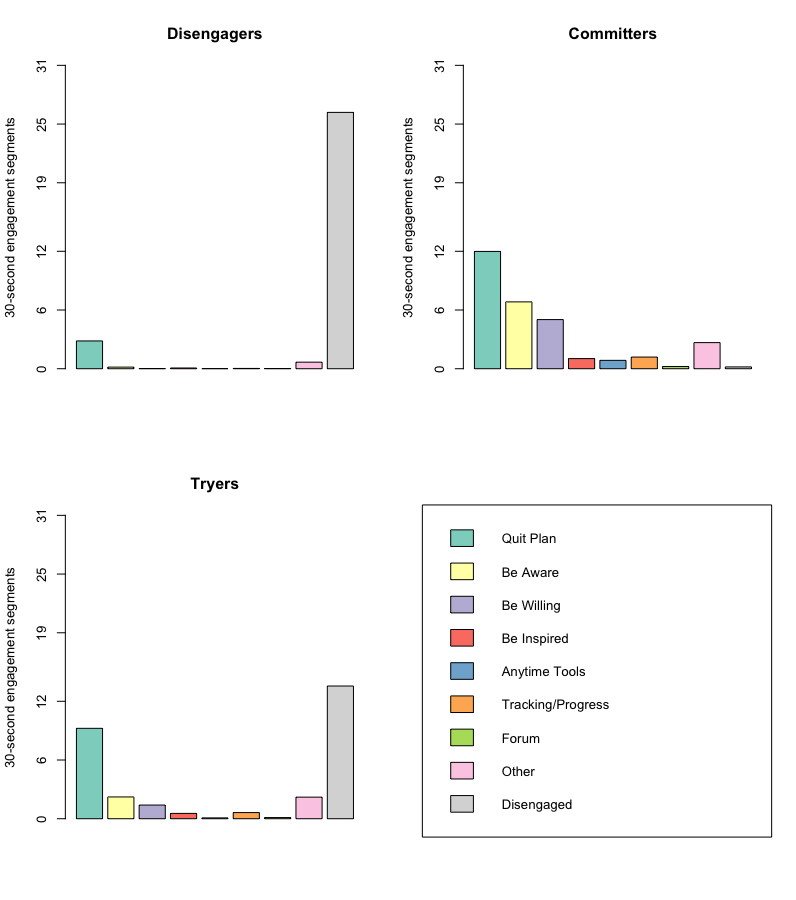
*

*f) The point at which 25% of participants had stopped engaging with the program used as cut-off*

*
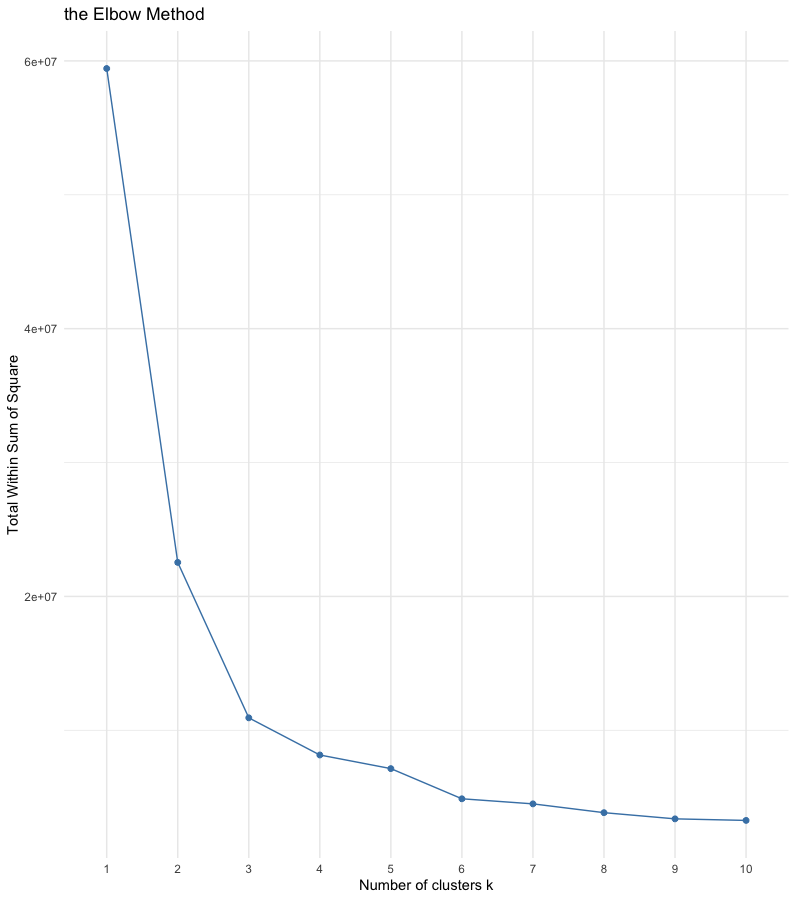
*

*
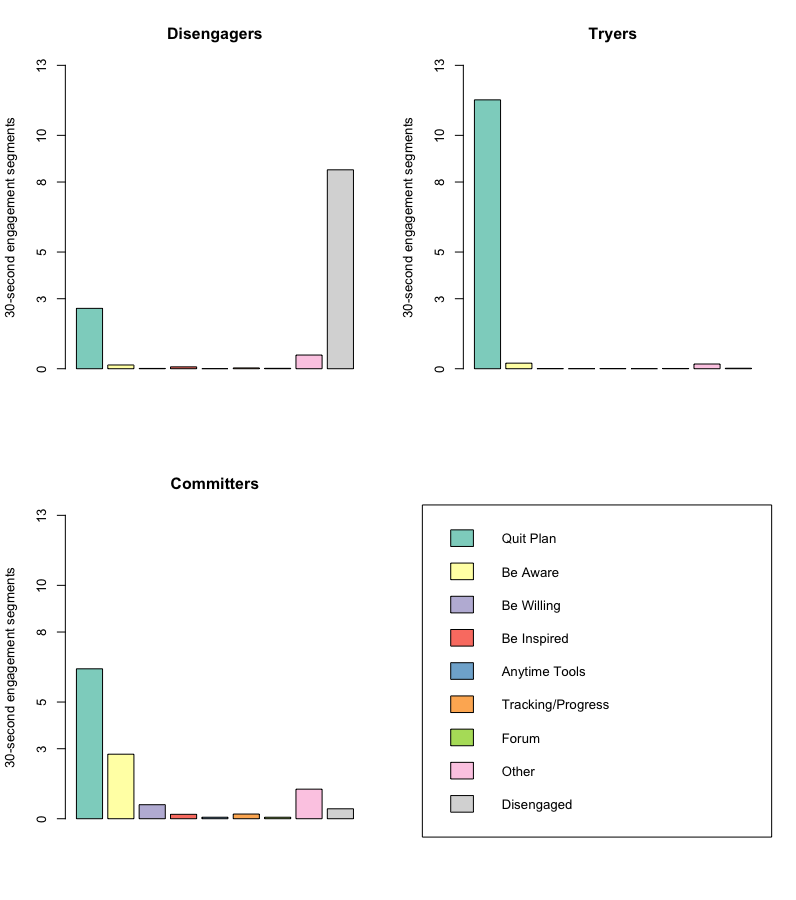
*
